# Tubercular Costochondritis Presenting as Chest Wall Swelling: A Case Report of an Atypical Tuberculosis Presentation

**DOI:** 10.7759/cureus.68158

**Published:** 2024-08-29

**Authors:** Aneeqa Qureshi, Ayesha Nazeef, Huzafa Ali, Jeevan Gyawali, Nadia Subhan

**Affiliations:** 1 Radiology, Aga Khan University Hospital, Karachi, PAK; 2 Internal Medicine, Quaid-e-Azam Medical College, Bahawalpur, PAK; 3 Internal Medicine, CMH Multan Institute of Medical Sciences (CIMS), Punjab, PAK; 4 Internal Medicine, Patan Academy of Health Sciences, Kathmandu, NPL; 5 Internal Medicine, Peshawar Medical College, Peshawar, PAK

**Keywords:** antituberculous therapy, musculoskeletal tuberculosis, atypical tuberculosis, chest wall swelling, tb costochondritis

## Abstract

Costochondritis is an inflammatory condition of the costochondral junctions, rarely due to tuberculosis (TB). One-quarter of the world's population is affected by tuberculosis, while musculoskeletal tuberculosis accounts for only 1-2% of the total cases. Among these cases, the involvement of the ribs is extremely rare.

The following case report describes a 60-year-old male with diabetes who had recurrent thoracic wall swelling with greenish discharge for 23 years, misdiagnosed and treated as sebaceous cysts. Recently, at its exacerbation, imaging and biopsy revealed tubercular costochondritis, a very rare form of extrapulmonary tuberculosis that affects the ribs. Antituberculous therapy administered for nine months showed complete resolution of symptoms.

This case underscores the key issue of placing tuberculosis within the differential diagnosis for a chest wall swelling, highlighting its diagnostic challenge in this atypical presentation. Advanced imaging and histological examination were of importance in coming up with an accurate diagnosis; hence, clinical suspicion needs to be increased and more research done in the light of management guidelines for this rare condition.

## Introduction

Tuberculosis, an infectious and communicable disease caused by the bacillus Mycobacterium tuberculosis, affects about a quarter of the world's population. Musculoskeletal tuberculosis is the most common form of extra-pulmonary tuberculosis, making up 1-2% of all tuberculosis cases and 10-15% of all extra-pulmonary tuberculosis in endemic regions. In skeletal system tuberculosis, the vertebral column is the most commonly affected site (50%), followed by the hip (15%) and knees (5%) [1].

Chest wall tuberculosis is a rare form of extra-pulmonary tuberculosis (EPTB), accounting for 1%-5% of all musculoskeletal tuberculosis cases [2]. Many national and international efforts to control tuberculosis have been made, but evidence shows most multidrug-resistant tuberculosis (MDR-TB) cases remain undetected and untreated, especially in developing countries [3]. Besides the spine, tuberculosis can affect sacroiliac joints, the ribs, and the sternoclavicular joints [4]. 

Systematic screening in areas with high tuberculosis prevalence is recommended for early disease detection. Effective antibiotic treatment is available for tuberculosis, highlighting the importance of early detection to reduce mortality and morbidity [5]. Thoracic wall tuberculosis typically presents gradually with pain and swelling. However, diagnosing chest wall TB is often delayed, and less than 50% of chest wall TB patients may have active pulmonary tuberculosis [6].

This is a rare case of tubercular costochondritis with no features of tuberculosis, only presenting with chest wall swelling. It presented diagnostic challenges that were somewhat intricate but meticulously worked out through an interdisciplinary approach and evidence-based practice that took central consideration for atypical presentations in the differential diagnoses.

## Case presentation

A 60-year-old male, non-smoker with diabetes mellitus, presented to the hospital for evaluation of a swelling on the right anterior thoracic wall, which had been present for 23 years. In 2010, the swelling discharged greenish, odorless, painless watery fluid, which was drained by a general surgeon and diagnosed as a sebaceous cyst. Over the past 13 years, the watery discharge recurred every six months to a year, lasting one to two days each time. Recently, the patient noticed another bulge lateral to the original one with a similar greenish, painless, odorless discharge, prompting his visit to the hospital. On general examination, the patient was afebrile and well-nourished with no history of weight loss. Local examination revealed a bulge with greenish discharge on the right anterior thoracic wall at the level of the fifth rib. The area was slightly tender, but there were no signs of peripheral lymphadenopathy. The abdomen was soft and non-tender, with no organomegaly. The overlying skin appeared normal, with no wounds, scars, rash, or sinuses.

Investigations revealed normal blood hemograms, blood coagulation tests, kidney function tests, and liver function tests. Mean corpuscular hemoglobin concentration (MCHC) was slightly raised to 36.3 g/dl (normal is 30-35.5 g/dl), and erythrocyte sedimentation rate (ESR) was high (19 mm/first hour; normal is 0-15 mm/first hour). The result for the interferon-gamma release assays (IGRAs) test was positive for infection with Mycobacterium tuberculosis, but Ziehl-Neelsen smear microscopy and sputum GeneXpert were negative. His glycated hemoglobin (HbA1c) was 7.2%, for which he was treated with metformin twice a day.

**Figure 1:**
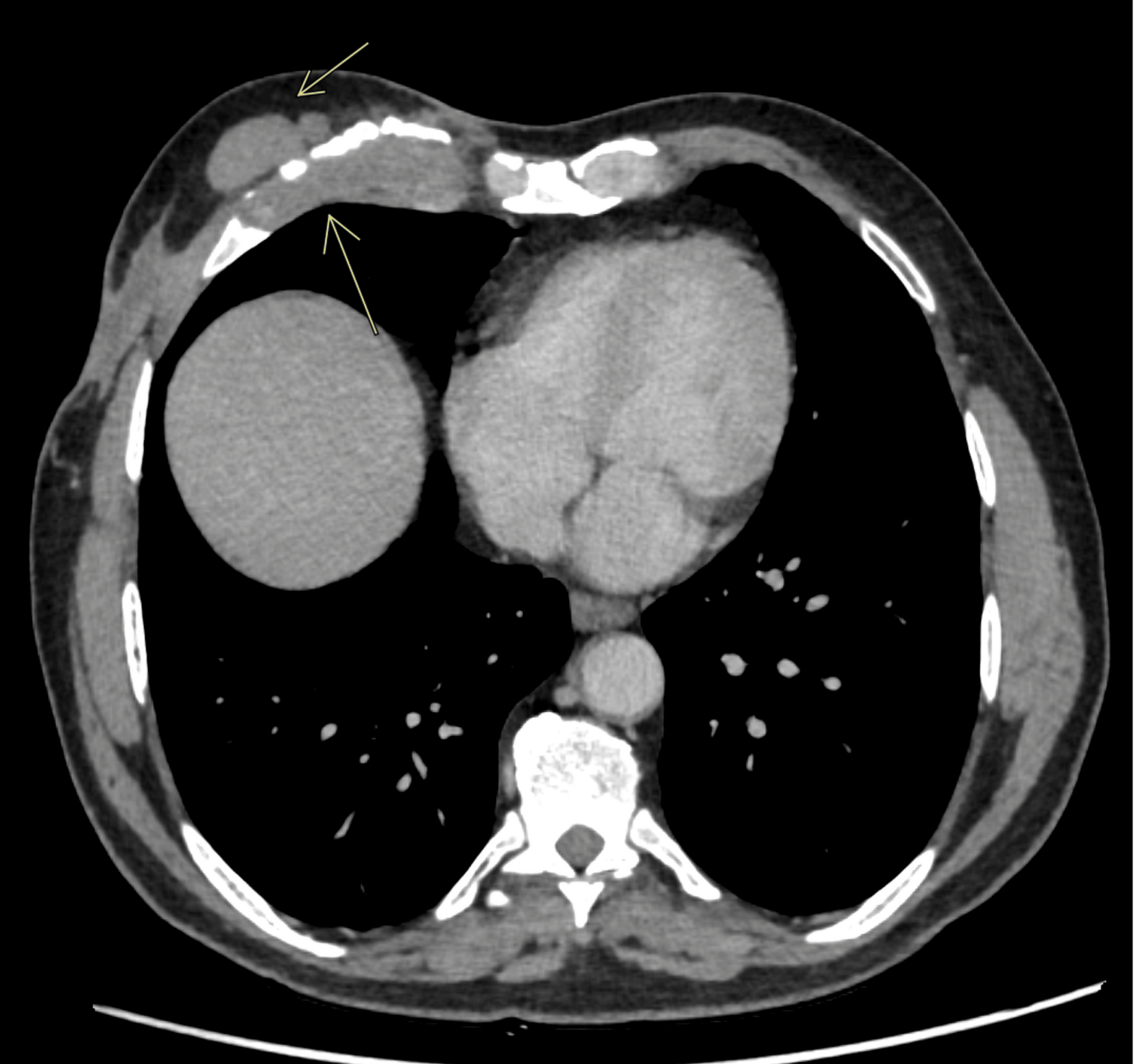
Contrast-enhanced computed tomography (CECT) chest findings Demonstrates soft tissue mass around the right fifth costal cartilage and bony erosion with fragmentation of cartilage. Well-defined soft tissue nodules were also noted in the anterior abdominal wall, as shown by the arrow.

**Figure 2:**
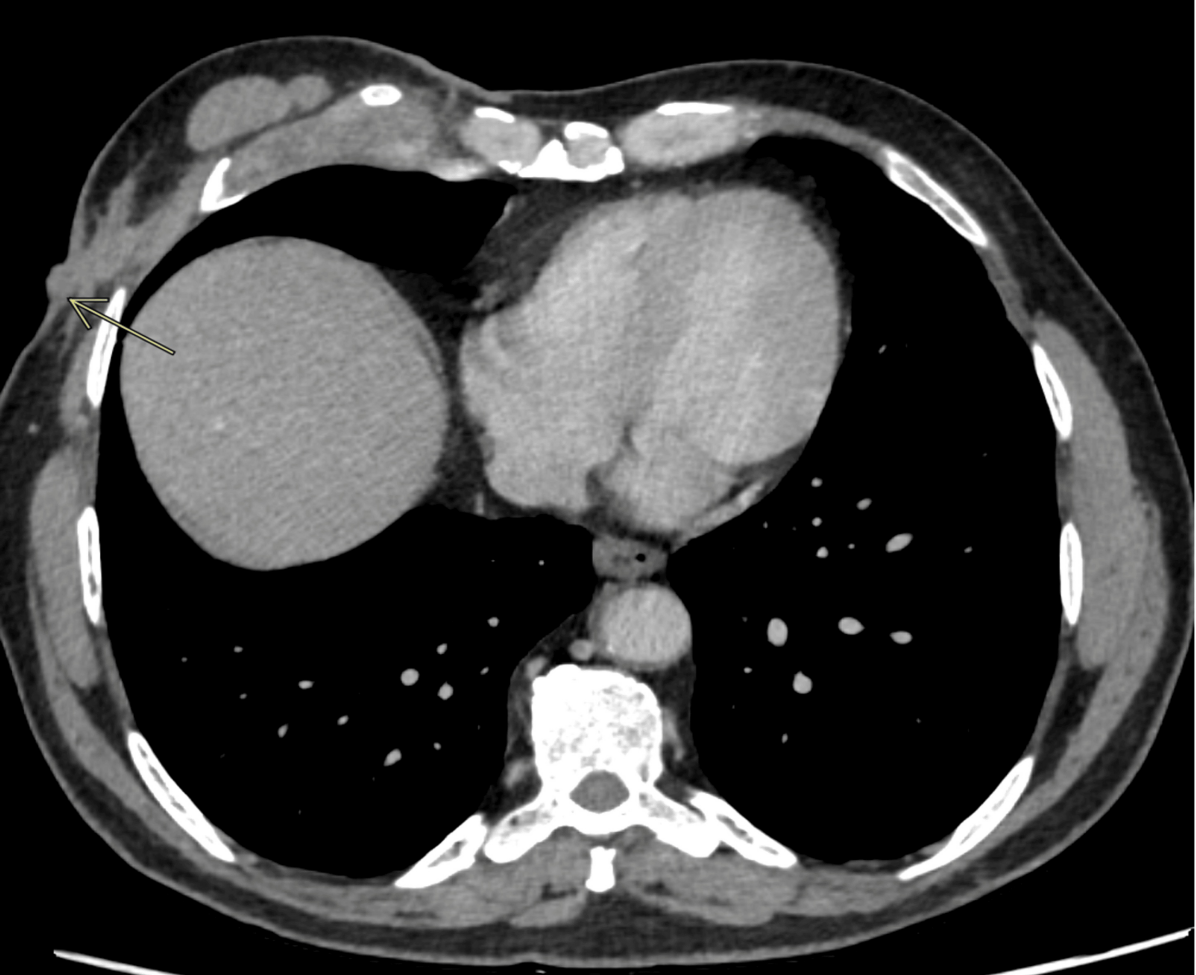
Contrast-enhanced computed tomography (CECT) chest findings Demonstrates a small linear hypodense fluid density area with 21 HU, with an external opening suggestive of a sinus tract.

**Figure 3:**
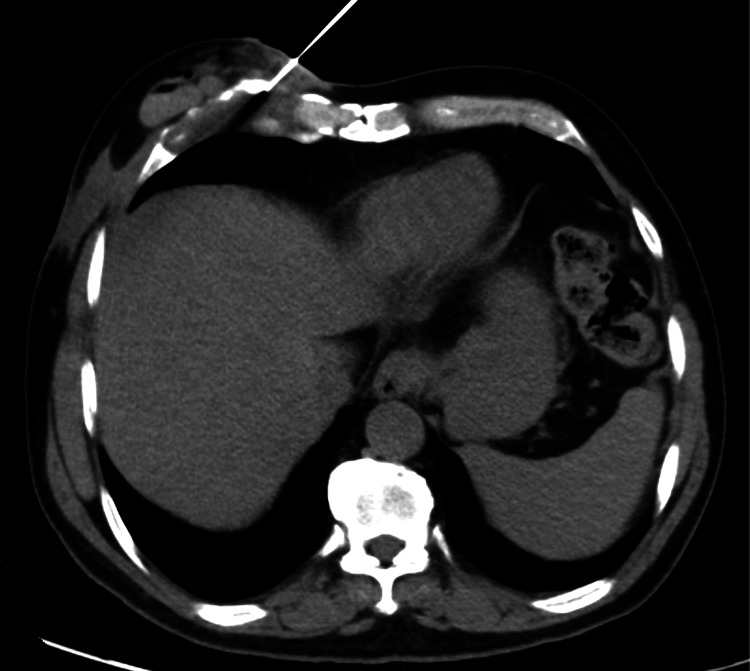
Contrast-enhanced computed tomography (CECT) guided biopsy of the right fifth costochondral junction

Axial computed tomography (CT) chest with contrast showed thickening at the costochondral junction of the right fifth rib concluding costochondritis (Figure 1), a small abscess with sinus tract (Figure 2) in the subcutaneous soft tissues of the anterior lateral chest wall (33 x 8 mm), along with multiple bilateral small granulomas, and a few well-defined ovoid soft tissue density nodules seen (in close proximity with each other) within the subcutaneous tissues of the anterior chest wall, likely representing neurofibromas. A biopsy was done with specimens taken from subcutaneous chest wall and rib lesions (Figure 3). Particularly rib lesions showed linear cores of tissue exhibiting fibrous stroma with areas of hyaline cartilage and moderately dense inflammatory infiltrate comprising lymphoplasmacytic cells, histiocytes, and few neutrophils. A well-defined granuloma is appreciated on levels with giant cells. A chest wall lesion biopsy revealed mostly necrotic tissue with viable regions exhibiting sclerotic stroma, vessels, and focal inflammatory cells. The conclusion pointed towards tubercular costochondritis.

The patient was started on antituberculous therapy, consisting of two months of isoniazid, rifampicin, pyrazinamide, and ethambutol, followed by ten months of isoniazid and rifampicin, as per the 2019 National Guidelines for TB of Pakistan [7]. Followup was maintained, and the patient's symptoms improved, drainage stopped, and the swelling resolved.

## Discussion

Tuberculosis remains one of the major public health problems throughout the world. Diagnosis of tuberculosis is often difficult because it is a great mimic with unusual clinical presentations and a variable course of illness. Though the spine is a common site for bone tuberculosis, any bone or joint in the human body may be involved [8].

Typical symptoms of bone tuberculosis include localized pain and swelling, often associated with systemic signs like low-grade fever, night sweats, appetite loss, and weight loss, which are hallmark clinical features of musculoskeletal tuberculosis [9]. Also, it can present with possible complications such as secondary infections, spontaneous fractures, or blood vessel compression [10]. Poverty, undernutrition, smoking, and immunocompromised conditions like human immunodeficiency virus (HIV), diabetes mellitus, and end-stage renal disease (ESRD) are the primary risk factors for tuberculosis infection [11].

The roentgenographic findings for rib tuberculosis were classified into four categories based on the affected rib part: costovertebral (35%), costochondral (13%), shaft (61%), and multiple cystic bones. In our case, the costochondral junction was affected. Multifocal rib involvement is also seen, though it is more common in immunocompromised patients. Proposed mechanisms for rib tuberculosis include hematogenous or lymphatic dissemination following the reactivation of dormant tuberculosis focus, the most common etiology, direct extension from mediastinal lymph nodes, or direct extension from the lungs [12]. 

A CT scan is more sensitive in these areas to identify soft tissue abnormalities and osseous destruction [13]. Axial CT chest with contrast in our case revealed thickening at the right fifth rib's costochondral junction and a tiny abscess in the subcutaneous soft tissues of the anterior and lateral chest walls, which may indicate costochondritis. Because it can be difficult to differentiate a chest wall abscess from a pyogenic abscess or chest wall tumor, diagnosing one can be difficult. The most frequent lumps in the chest wall are called soft tissue tumors, and they play a key role in the differential diagnosis [14]. In our case, the result of the biopsy revealed well-defined granulomas and giant cells, and a positive IGRAs test confirmed the diagnosis of tubercular costochondritis.

The optimal treatment for chest wall tuberculosis remains uncertain. Further research is needed to determine whether antitubercular therapy alone or surgical debridement (or excision based on lesion extension) in combination with antitubercular therapy is more effective. However, the general guideline suggests that if complications arise, surgery is preferred, followed by antitubercular therapy [4].

The case report has its limitations in that it is a single case, thus generalization may not be possible, and lack of long-term follow-up data has limited understanding of prognosis and outcome. There is an inherent selection bias in reporting unique cases, which may overemphasize the actual clinical significance. In addition, culture for Mycobacterium tuberculosis could not be done.

## Conclusions

This case report highlights a rare instance of tubercular costochondritis, which appeared only with chest wall swelling and presented huge diagnostic difficulty. Thus, an interdisciplinary approach and evidence-based practice were very important when considering atypical presentations. There are complexities in the management of tubercular costochondritis because the natural course of the disease is quite unpredictable, and further studies on the subject are required to determine proper treatment policies, especially those related to surgical intervention. Although it suffers from limitations regarding generalisability and long-term follow-up, this case improves our understanding of such rare presentations of tuberculosis and does reiterate that early identification and timely interventions can ensure better outcomes for the patients.
